# Clinical course after the onset of choroidal neovascularization in eyes with central serous chorioretinopathy

**DOI:** 10.1097/MD.0000000000026980

**Published:** 2021-08-27

**Authors:** Rae-Young Kim, Gun-Jung Ma, Woo-Kyung Park, Mirinae Kim, Young-Gun Park, Young-Hoon Park

**Affiliations:** a Department of Ophthalmology and Visual Science, Seoul St. Mary's Hospital, College of Medicine, The Catholic University of Korea, Banpo-daero, Seocho-gu, Seoul, Korea; b Catholic Institute for Visual Science, College of Medicine, The Catholic University of Korea, Seoul, Korea.

**Keywords:** central serous chorioretinopathy, choroidal neovascularization, prognosis, visual acuity

## Abstract

Chronic central serous chorioretinopathy (CSC) can be complicated with choroidal neovascularization (CNV); however, the timing of its occurrence and its clinical significance are not well understood. This study aimed to observe the time of choroidal neovascularization detection after CSC diagnosis and determine whether clinical features and prognosis differed in patients with chronic CSC or age-related retinal degeneration.

In this retrospective study, medical records of CSC patients complicated with CNV who visited Seoul St. Mary's hospital of Korea between October 2009 and December 2020 were reviewed. The presence of CNV was determined using fluorescein, indocyanine green, or optical coherent tomography angiography (OCTA). Based on the patients’ medical records, we observed the change of clinical pattern, best-corrected visual acuity (BCVA) and central macular thickness (CMT) at CNV detection and at 6 months, 1 year, 3 years, and 5 years following CNV detection.

Thirty eyes of 30 patients (male: female ratio of 13:17) were enrolled. Mean age at diagnosis of CSC was 54.0 ± 8.5 years (mean ± standard deviation). On average, CNV was detected 1.65 ± 2.30 years after the diagnosis of CSC. The mean CMT was significantly decreased at 6 months, 1 year, and 3 years after choroidal neovascularization detection (*P* < .001, *P* < .001, *P* = .001 respectively). BCVA tend to improve after CNV detection, but there was no statistical significance at 6 months, 1 year, 3 years, and 5 years (all with *P* > .05). There were no clinical findings suggesting age-related macular degeneration such as intraretinal, subretinal hemorrhage or drusen in any of the case during follow-up. None of the subjects had severe visual acuity loss of 1.0 logarithm of the minimum angle of resolution (logMAR) (20/200 Snellen equivalent) or greater. Among the subjects, 6 patients (20%) did not require any treatment during observation, while 24 other patients required anti-vascular endothelial growth factor (anti-VEGF) or photodynamic therapy. At the last visit, 22 patients (73.3%) remained stable for more than 6 months, without subretinal fluid recurrence.

Choroidal neovascularization was detected earlier than previously reported. There was no rapid deterioration of visual acuity or clinical features even after CNV detection.

## 1 Introduction

Central serous chorioretinopathy (CSC) is a posterior segment eye disease characterized by serous sensory retinal detachment at the macular region, with unresolved pathogenic mechanisms.^[1–3]^ The natural course of CSC is highly heterogeneous among patients and it is typically classified as acute or chronic.^[4,5]^ Generally, acute CSC resolves spontaneously within 3 to 4 months, unlike chronic CSC, which persists for more than 6 months. Further, chronic CSC is characterized by atrophy in the extended retinal pigment epithelium (RPE) area. Persistent choroidal congestion due to chronic CSC may lead to the attenuation of the inner choroid, resulting in the development of complications, such as retinal atrophy and choroidal neovascularization (CNV).^[1]^

CNV is a known complication of CSC. Previous studies have reported that its prevalence rates vary from 2% to 84%.^[1,6,7]^ Mrejen et al recently reviewed 217 eyes from 133 patients with chronic CSC using fluorescein angiography (FA) and indocyanine green angiography (ICGA). They reported that 52 eyes (24%) of 37 patients had CNV, as a complication of CSC, with the mean detection time being 17.0 ± 10.4 years after the first visit to an ophthalmologist.^[8]^

It can be challenging to differentiate CSC from age-related macular degeneration (ARMD) using FA and ICGA, especially when CSC is combined with CNV. These 2 conditions may have very similar features on FA/ICGA.^[9]^ Recently, the advancement of optical coherence tomography angiography (OCTA) imaging has enabled its use as a noninvasive tool in the visualization of CNV. According to a study based on patients with CSC having flat irregular pigment epithelium detachment (FIPED), OCTA imaging detected the presence of CNV in 35.6% of patients. Conversely, CNV was detected in only 25% of cases with FIPED using a combination of spectral-domain optical coherent tomography (OCT), FA, and ICGA.^[10]^

Shiragami et al reported that the chronicity of CSC was significantly associated with CNV.^[11]^ However, the duration of CNV detection after the initial diagnosis of CSC has not been sufficiently investigated. Moreover, long-term visual prognosis and changes in the clinical features of CSC after CNV detection have not been clearly defined. Chronic CSC causes widespread RPE and choriocapillaris changes and are more likely to be complicated with CNV, resulting in a devastating effect on visual acuity.^[12]^ Therefore, the diagnosis of CNV in patients with CSC is critical. As mentioned earlier, several studies have reported that chronic CSC could be complicated with CNV.^[1,6–8]^ However, the timing of its occurrence and its clinical significance are not well understood. Therefore, we aimed to observe the duration from CSC diagnosis to CNV occurrence and examine whether the clinical characteristics and prognosis in patients with chronic CSC after CNV detection are different from those reported in previous studies.

## 2 Methods

This study was conducted in the Department of Ophthalmology of Seoul St. Mary's Hospital, a tertiary hospital in Korea and followed the tenets of the Declaration of Helsinki. All protocols were approved by the Catholic University of Korea, Seoul St. Mary's Hospital, Institutional Review Board (KC20RISI0956). The requirement for informed consent was waived because of the retrospective longitudinal design of this study.

### 2.1 Participants

All participants were assessed between October 2009 and December 2020 at Seoul St. Mary's Hospital in Korea. The analysis of medical records and imaging data was performed retrospectively on cases complicated with CNV among patients diagnosed with CSC. Patients diagnosed with CSC who underwent adequate ocular examination and had sufficient medical records were included in our study. The medical records and imaging data of patients diagnosed with CNV as a complication of CSC were retrospectively reviewed by 2 retinal specialists (YGP, YHP) at the clinic of the Vitreoretinal Department of Seoul St. Mary's Hospital in Korea. Cases that had already been diagnosed with exudative ARMD or polypoidal choroidal vasculopathy at the start of the follow-up observation period were excluded. In addition, patients with retinal vascular occlusion, diabetic retinopathy, and macular hole or neurodegenerative diseases, previous vitrectomy, uveitis, uncontrolled glaucoma history were excluded from this study. Patients who underwent photodynamic therapy (PDT) and focal laser photocoagulation before CNV detection were excluded from the study because of the possibility of developing laser induced secondary CNV. Moreover, patients with other systemic diseases that could affect the eye and media opacity and the image quality were also excluded from the analysis.

### 2.2 Study protocol

We reviewed the patients’ medical records. Demographic data, including sex, age at initial diagnosis of CSC, and age at CNV detection, were collected. Medical information, including a history of hypertension (HTN), diabetes mellitus (DM), ocular comorbidities, and a history of ocular surgery and treatment for CSC, was recorded for analysis. Ocular examination records, including the best-corrected visual acuity (BCVA) assessment through the Snellen chart, non-contact pneumatic tonometry, slit-lamp biomicroscopy, and dilated fundus examination, were also collected.

When a patient was diagnosed and treated at another ophthalmologic clinic prior to referral to our clinic, which is a tertiary medical institution, the dates of diagnosis of CSC was defined according to the attached prior medical records. The patients received anti-vascular endothelial growth factor (anti-VEGF) therapy when the subretinal fluid (SRF) due to CSC lasted more than 3 months without improvement according to the clinicians’ decision. PDT was selectively performed in patient without response in multiple anti-VEGF therapy. After 1 month of treatment, the treatment response was evaluated according to the change in the subretinal fluid. Good response to the treatment was defined as complete loss of SRF. Increases or no definite change in subretinal fluid was defined as no response to treatment. If the subretinal fluid decreased but was not completely lost, it was defined as a partial response to the treatment.

### 2.3 Image analysis

Multimodal imaging data including fundus photography, FA and ICGA (Heidelberg Spectralis, Heidelberg, Germany), OCT, and OCTA were acquired. All patients had at least 4 visits during the clinical course of their disease.

Macular OCT evaluations were performed using the Heidelberg Spectralis HRA-OCT (Heidelberg Engineering, Germany; volume scan between 20° × 15° and 30° × 25° in dimensions). Enhanced depth imaging OCT scans (Heidelberg Engineering, Germany) over a 30° × 5° region of the central macula, with 100 images averaged using 7 sections, were used to evaluate the choroidal features. To obtain OCTA images, Topcon OCTA device (Topcon Corporation, Japan) was used. The scanning area was captured in 3 × 3 mm sections centered on the fovea.

CSC was defined by its clinical manifestations and the presence of serous sensory retinal detachment at the macular region, with or without pigment epithelial detachment (PED) using OCT. When the symptom duration was greater than 6 months on consecutive visits or when recurrent episodes were noted, it was classified as chronic CSC. When the symptoms self-resolved without treatment within 6 months, it was classified as acute CSC.

**Figure 1:**
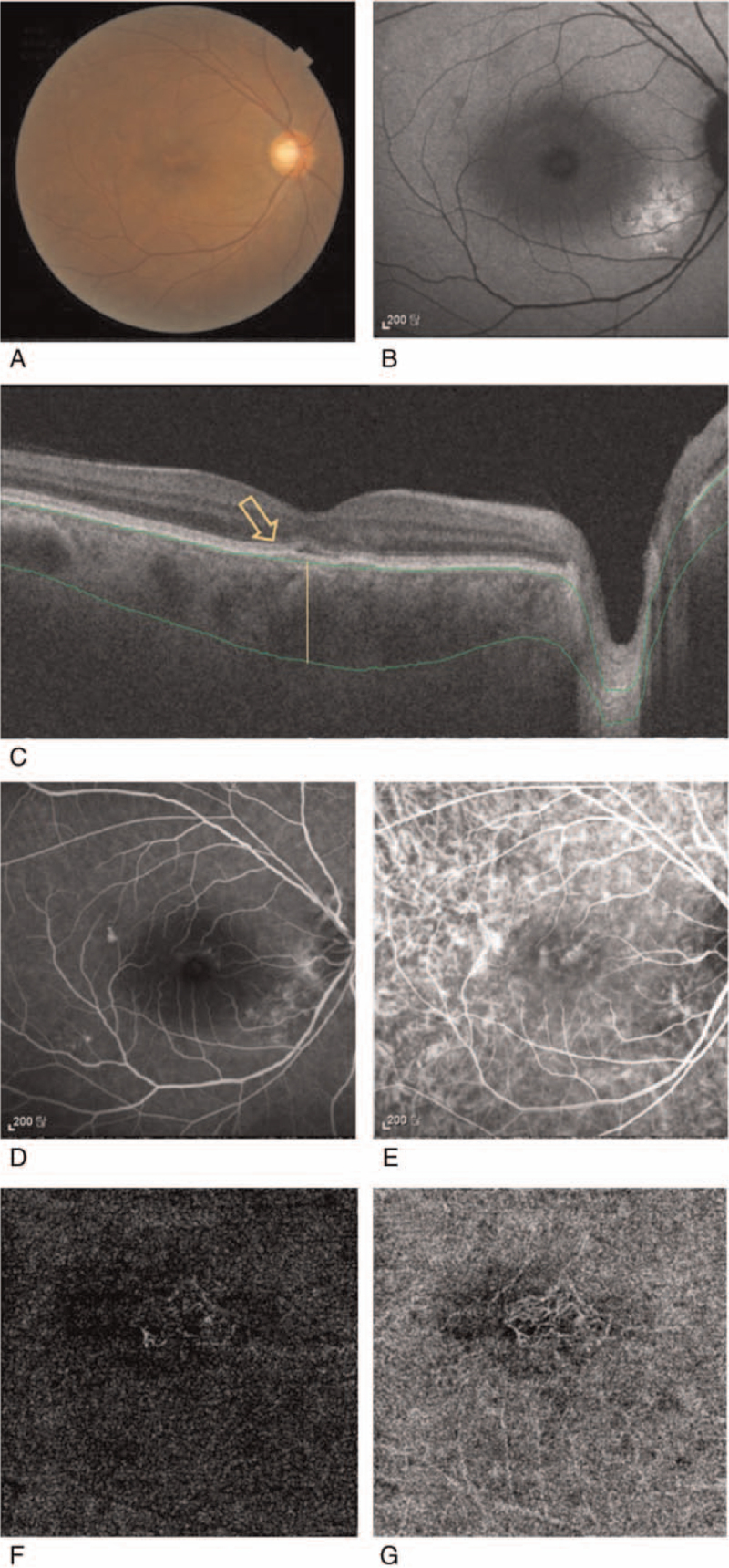
Representative figure of CSC with CNV. The fundus of a 63-year-old male patient was diagnosed with chronic CSC in his right eye (A). Retinal pigment epithelium (RPE) change was found at the inferonasal side of the macular region (B). Minimal serous sensory retinal detachment with flat RPE detachment (arrows) at the macular region was detected by swept-source OCT. A thicker subfoveal choroid with congestion (yellow line) was also found. The green lines represent the RPE layer and the chorioscleral interface (C). Fluorescein angiography shows focal hyperfluorescence at the macular lesion (D). Indocyanine green examination shows hypercenescence in the same area, which may represent choroidal congestion (E). No definite feature of the active leaking form of CNV can be observed. (F) CNV corresponding to the sub-RPE elevation region is found by OCT angiography in the outer retinal slab and (G) choriocapillaris slab. CNV = choroidal neovascularization, CSC = central chorioretinopathy, OCT = optical coherent tomography, RPE = retinal pigment epithelium.

The presence of CNV was determined using angiographic findings and OCTA, combined with OCT imaging. CNV was defined as FIPED with hyper-reflective or heterogeneous reflective findings in the sub-RPE space on OCT, a CNV network in the outer retina and choriocapillaris slab on OCTA, ill-defined late leakage on FA, and a late staining pattern on ICGA (Fig. 1), as described by Shiragami et al.^[11]^ A definite CNV network observed in the early phase of FA or ICGA was determined as CNV, according to Mrejen et al^[8]^ CNV was classified into types 1, 2, and 3, with or without a polypoidal choroidal vasculopathy component, using the Gass-Freund classification.^[13]^

Two independent retinal specialists (MK and WKP) performed all assessments of FA/ICGA, OCT, and OCTA images, and were blinded to the other imaging findings and the patients’ clinical history. Disagreements regarding the interpretation of images were resolved by open discussion between the 2 specialists.

Central macular thickness (CMT) was defined as the distance between the inner retinal surface and the inner border of the retinal pigment epithelium and measured automatically using the built-in software of the OCT devices.

### 2.4 Statistical analysis

Statistical analysis was performed using the Statistical Package for the Social Sciences (SPSS) for Windows version 24.0 (IBM Corp., Armonk, NY).

The sample size was calculated using the paired means power analysis. A sample size of 27 at 1 year achieved 84% power detect a mean paired differences of 0.1 logarithm of the minimum angle of resolution (logMAR) with an estimated standard deviation of difference of 0.2 and with a significance level (alpha) of 0.05 using a two-sided paired *t*-test. And at 3 years, a sample size of 17 achieved 74% power detect a mean paired differences of 0.1 logMAR with an estimated standard deviation of difference of 0.1 and with a significance level (alpha) of 0.05 using a two-sided paired *t*-test.^[14,15]^

An exploratory analysis was performed for all variables. The normality of data distribution was confirmed using the Kolmogorov-Smirnov test. The age at the onset of initial symptom and at CSC diagnosis satisfied normality. BCVA measurements were converted to the logMAR scale for statistical analysis. Fisher exact probability test was used for the participants’ categorical and ocular characteristics potentially associated with CNV (sex, HTN or DM status, and CSC type, whether chronic or acute). Measurements of BCVA, CMT, and subfoveal choroidal thickness at each follow-up time point (on CNV detection, at 6 months, and at 1 year) did not satisfy normality; hence, we used the Wilcoxon signed-rank test. We analyzed the repeated measures (BCVA, CMT) through time using a linear mixed regression model. Univariate and multiple linear regression analyses were performed to analyze the effects of multiple factors associated with BCVA at 6 months and 1 year after CNV diagnosis in patients with CSC. Data are presented as means ± standard deviation (SD) where applicable. Multivariate linear regression analysis of all factors included in the univariate and multivariate studies was performed. A two-sided *P* value of <.05 was considered statistically significant.

## 3 Results

### 3.1 Demographics and clinical characteristics

A total of 30 eyes from 30 patients (male: female ratio of 13:17) were analyzed in this study. There were bilateral CSC cases, but no case has CNV in both eyes. The mean age at the diagnosis of CSC was 54.0 ± 8.5 (mean ± SD) years. The average age at CNV detection was 55.7 ± 9.2 (mean ± SD) years. The demographic and systemic characteristics, the bilaterality of CSC, and the initial BCVA values of the participants are shown in Table 1. Two (6.7%) patients had a history of DM and 5 (16.7%) patients had a history of HTN. A total of 14 eyes (46.7%) of all participants had CSC in the left eye. The mean BCVA values were 0.15 ± 0.2 logMAR (Snellen equivalent of 20/28) at the initial visit.

**Table 1:** Summary of demographic and baseline clinical characteristics of all subjects.

Demographic information	Value
No. of eyes	30
Sex	
Female	17 (56.67%)
Male	13 (43.33%)
Age at diagnosis of CSC (yr)	54.0 ± 8.5
Age at CNV detection (yr)	55.7 ± 9.2
DM	2 (6.67%)
HTN	5 (16.67%)
OD/OS	16/14 (53.3%/46.7%)
Bilaterality of CSC	2 (6.67%)
Initial BCVA, logMAR with mean Snellen equivalent	0.15 ± 0.20, 20/28

Value of results are presented as mean ± standard deviation or number (%). BCVA = best-corrected visual acuity, CNV = choroidal neovascularization, CSC = central serous chorioretinopathy, DM = diabetes mellitus, HTN = hypertension, logMAR = logarithm of the minimum angle of resolution, OD = “oculus dexter”/right eye, OS = “oculus sinister”/left eye.

The clinical characteristics are summarized in Table 2. The mean follow-up duration from the initial visit to the last visit to our clinic was 3.76 ± 2.51 (mean ± SD) years and from CNV detection to last visit were 2.83 ± 1.63 (mean ± SD) years. The CNV was detected 1.65 ± 2.30 (mean ± SD) years on average after the diagnosis of CSC. One patient (3.3%) had acute CSC, while the rest (96.7%) had chronic CSC. The mean subfoveal choroidal thickness at the point of diagnosis of CNV was 372.27 ± 128.27 μm. All cases of CNV were classified as type 1, and only 2 eyes (6.7%) had CNV combined with polyp-like appearance. CNV was detected using FA/ICGA in 8 (26.7%) patients, with OCTA in 17 (56.7%) patients, and through FA/ICGA with OCTA in 5 (16.7%) patients. There was no active vascular leakage at the choroidal neovascular network or a polyp-like structure in dye angiography.

**Table 2:** Summary of follow-up period, type of central serous chorioretinopathy, and characteristics of the choroid.

Characteristics	Total (N = 30)
Follow-up period	
From the initial visit to the last follow-up (yrs)	42.36 ± 35.12
From the CNV detection to the last visit (yrs)	3.76 ± 2.51
From the diagnosis of CSC to CNV detection (yrs)	2.83 ± 1.63
Type of CSC	
Acute	1 (3.33%)
Chronic	29 (96.67%)
Choroid	
Initial SFCT (μm)	372.27 ± 128.27
Type of CNV	
Type 1 CNV	28 (93.33%)
Type 1 combined with polyp-like structure	2 (6.67%)

Value of results are presented as mean ± standard deviation or number (%). CNV = choroidal neovascularization, CSC = central serous chorioretinopathy, SFCT = subfoveal choroidal thickness.

### 3.2 Changes in best-corrected visual acuity and central macular thickness

**Figure 2:**
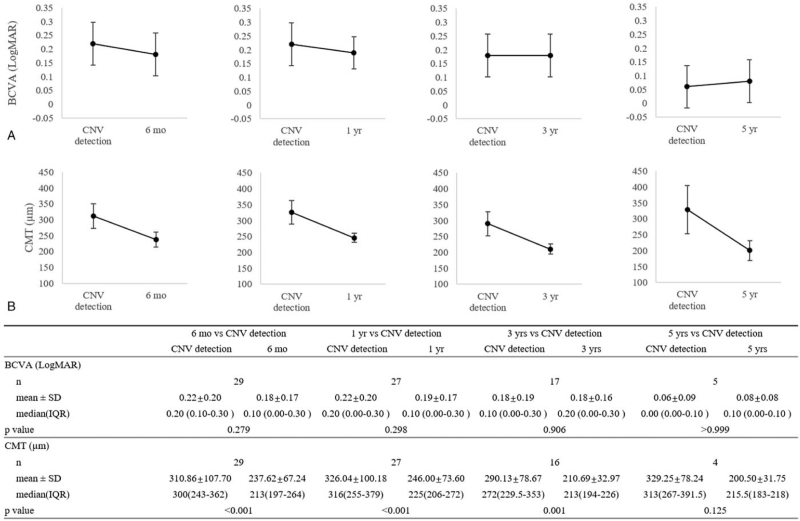
Best-corrected visual acuity and central macular thickness at the time of choroidal neovascularization detection, 6 months, 1 year, 3 years, and 5 years after choroidal neovascularization detection. The graph represents mean value at each time point. The *P* values for differences were determined using the Wilcoxon signed rank test for paired data. The 4 graphs in the top row show that BCVA tend to improve during follow-up after CNV detection except 5 years, but there was no statistical significance. (All *P* > .05). The 4 graphs in the bottom row show that the mean CMT was significantly decreased at 6 months, 1 year, and 3 years after choroidal neovascularization detection (*P* < .001, *P* < .001, *P* = .001 respectively). There was no significant difference at 5 years. (*P* = .125). BCVA = best-corrected visual acuity, CMT = central macular thickness, CNV = choroidal neovascularization, logMAR = logarithm of the minimum angle of resolution.

We collected the data of BCVA and CMT for a period of 5 years from CNV detection in patients with CSC (Fig. 1). The mean BCVA values were 0.22 ± 0.20 (logMAR; n = 30; Snellen equivalent, 20/33) at the point of CNV detection, 0.18 ± 0.17 (logMAR; n = 29; Snellen equivalent, 20/30, *P* = .279) after 6 months, 0.19 ± 0.17 (logMAR; n = 27; Snellen equivalent, 20/31, *P* = .298) after 1 year, 0.18 ± 0.16 (logMAR; n = 17; Snellen equivalent, 20/31, *P* = .906) after 3 years, and 0.08 ± 0.08 (logMAR; n = 5; Snellen equivalent, 20/25, *P* > .999) after 5 years of CNV detection. At each follow-up point, mean BCVA values were better than those at the time of CNV detection, but there was no statistical significance (all *P* > .05) (Fig. 2).

The mean CMT was 310.83 ± 105.82 μm (n = 30) at the time of CNV detection. The mean follow-up CMT from CNV detection was decreased to 237.62 ± 67.24 μm (n = 29, *P* < .001) after 6 months, 246.00 ± 73.60 μm (n = 27, *P* < .001) after 1 year, 210.69 ± 32.97 μm (n = 16, *P* = .001) after 3 years, and 200.50 ± 31.75 μm (n = 4, *P* = .125) after 5 years (Fig. 2). To take into consideration the repeated measures through time, we performed analysis using linear mixed regression model (Table 3). BCVA change was not significantly changed over time and each time point (all *P* > .05). And CMT change was significantly decreased over time and each time point (all *P* < .05). To analyze the factors affecting the follow-up BCVA, we performed a univariate analysis. Univariate regression analysis revealed that the age at diagnosis, at initial symptom onset, and at CNV detection, sex, and HTN or DM status were not significantly correlated with the BCVA at 6 months and 12 months after CNV detection. Only BCVA values at CNV detection were significantly correlated with BCVA at 6 months (*P* = .039), and the initial BCVA was significantly correlated with BCVA at 12 months (*P* = .029) (Table 4). Multivariate linear regression analysis of all factors included in the univariate analysis revealed that no factor was significantly associated with BCVA at 6 months and 12 months after CNV detection.

**Table 3:** The trend of changes in best corrected visual acuity and central macular thickness with time, based on a linear mixed regression model.

							Bonferroni post hoc test of the *P* value			
	CNV detection	6 month	1 year	3 years	5 years	*P* value of changes over time	6 month vs CNV detection	1 year vs CNV detection	3 years vs CNV detection	5 years vs CNV detection
BCVA (LogMAR)										
n	30	29	27	17	5					
mean ± SE	0.23 ± 0.04	0.18 ± 0.03	0.19 ± 0.03	0.18 ± 0.04	0.08 ± 0.04	0.491	0.288	> 0.999	>0.999	>0.999
CMT (μm)										
n	30	29	27	16	4					
mean ± SE	310.83 ± 19.32	237.62 ± 12.49	246.00 ± 14.16	210.69 ± 8.24	200.50 ± 15.88	< 0.001	<0.001	<0.001	<0.001	0.003

A linear mixed regression model with time as fixed effects, random intercept, and an unstructured covariance matrix, pairwise comparisons were adjusted for using Bonferroni's procedure to account for multiple testing. 1 yr = 1 year, 3 yrs = 3 years, 5 yrs = 5 years, 6 mo = 6 months, BCVA = best-corrected visual acuity, CMT = central macular thincess, CNV = choroidal neovascularization, logMAR = logarithm of the minimum angle of resolution, vs = versus.

**Table 4:** Univariate and multivariate analysis of factors associated with best corrected visual acuity at one year follow-up.

	BCVA at 12 months (logMAR)			
	Univariate analysis		Multivariate analysis	
Variables	Beta coefficient	Significance	Beta coefficient	Significance
Age at CSC diagnosis	0.004	0.340	0.015	0.229
Age at symptom onset	0.004	0.340	0.088	0.198
Age at CNV diagnosis	0.002	0.556	−0.099	0.183
HTN	0.070	0.521	0.031	0.818
DM	−0.258	0.057	−0.170	0.300
Sex	0.074	0.300	0.107	0.188
Symptom onset to CNV	−0.002	0.435	0.003	0.407
Diagnosis to CNV	−0.002	0.289	0.003	0.705
BCVA at CNV detection (logMAR)	0.266	0.123	0.163	0.579
BCVA at the initial visit (logMAR)	**0.356**	**0.029** ^∗^	0.089	0.790

∗ *P* < .05. BCVA = best-corrected visual acuity, CNV = choroidal neovascularization, CSC = central serous chorioretinopathy, DM = previous history of diabetes, HTN = previous history of hypertension, logMAR = logarithm of the minimum angle of resolution.

Most patients showed stable BCVA 1 year and 3 years after CNV detection as shown in Table 5. At 1 year, 24 out of 28 eyes (85.7%) remained in the same visual acuity category, 1 eye (3.6%) had worse visual acuity and 3 eyes (10.7%) had improved visual acuity. At 3 years, 16 out of 17 eyes (94.1%) showed no change in visual acuity and other 1 eye (5.9%) had worsened visual acuity. No case with severe deterioration of BCVA exceeding 1.0 (logMAR) was found at 1 year and at 3 years after CNV detection.

**Table 5:** Frequency of best-corrected visual acuity change between at the point of choroidal neovascularization detection and 1-, 3-year thereafter.

	BCVA at 1-year follow up (n = 28)			
	logMAR ≤ 0.3	0.3<logMAR < 1	logMAR ≥ 1	Total
BCVA at CNV detection				
logMAR ≤ 0.3	21 (75.0)	1 (3.6)	0	22 (78.6)
0.3 < logMAR < 1	3 (10.7)	3 (10.7)	0	6 (21.4)
logMAR ≥ 1	0	0	0	0
Total	24 (85.7)	4 (14.3)	0	28 (100)

	BCVA at 3-year follow up (n = 17)			
	logMAR ≤0.3	0.3 < logMAR < 1	logMAR ≥1	Total
BCVA at CNV detection				
logMAR ≤0.3	14 (82.4)	1 (5.9)	0	15 (88.2)
0.3 < logMAR < 1	0	2 (11.8)	0	2 (11.8)
logMAR ≥1	0	0	0	0
Total	14 (82.4)	3 (17.6)	0	17 (100)

BCVA = best-corrected visual acuity, CNV = choroidal neovascularization, logMAR = the logarithm of the minimum angle of resolution, logMAR 0.3, 20/40 in Snellen equivalent; logMAR 1, 20/200 Snellen equivalent. Data are presented as counts, with the corresponding percentages in parenthesis.

### 3.3 Treatment response and long-term clinical course

Among 30 patients, observation was performed without treatment in 6 patients (20.0%). Fifteen patients (50.0%) were treated with anti-VEGF only and 9 patients (30.0%) who had no response to the anti-VEGF injection underwent PDT therapy. Of the 24 patients who received at least 1 anti-VEGF injection treatment, 10 (41.7%) had partial response, 9 (37.5%) had no response and only 5 (20.8%) patients had good response. All 9 patients who received PDT showed good response to PDT treatment and no recurrence was found after PDT.

At the last visit, 22 patients (73.3%) remained stable more than 6 months without SRF recurrence and treatment. In 6 patients (20.0%), there was no SRF, but they had recurrence of SRF within 6 months. Only 2 patients (6.7%) showed SRF. No intraretinal, subretinal hemorrhage or drusen was found in any of the case during follow-up.

## 4 Discussion

CNV is a major complication that can accompany CSC.^[16]^ As previously reported, the prevalence of CNV in patients with CSC ranges from 2% to 9%.^[1,6,7]^ In a study of 219 eyes, 144 had acute CSC while 58 eyes had chronic CSC. Among the 58 eyes with chronic CSC, 82.8% (48/58 eyes) had CNV complications. In another study that examined the long-term prognosis in 217 eyes with CSC, CNV was detected in 52 eyes.^[8]^ These differences in prevalence are associated with the difficulty in CNV detection in patients with CSC.

The etiology of CNV complicating CSC is not well known. Shiragami et al reported the clinical features and risk factors associated with neovascular CSC.^[11]^ Chronicity of the disease, female sex, choroidal vascular hyperpermeability, and poor BCVA values were suggested as significant risk factors for CNV in eyes with CSC in that study.^[11]^ Among these, CSC chronicity is an important risk factor that is possibly related to the pathophysiology of CNV, as it causes RPE and choriocapillaris changes. The main outcome of our study was the time to CNV detection, which was 1.65 ± 2.30 years from the first diagnosis of CSC. In a recent retrospective study, it was reported that during the follow-up, they detected 52 eyes (23.9%) with CNV among 217 eyes with chronic CSC using dye angiography (FA or ICGA). Further, the period from CSC diagnosis to CNV detection was reported to be 17.0 ± 10.4 years,^[8]^ which was considerably longer than the corresponding period in our study.

A previous study reported higher CNV incidence for women than for men and suggested that being female is possibly associated with the development of CNV in CSC.^[11]^ Moreover, in neovascular ARMD, incidence rates were higher in female than in male but the reason is not well known. It is thought to have hormonal etiology.^[17]^

Although the study by Mrejen et al did not clearly indicate the use of dye angiography, the representative figure of CNV in patients with CSC presented in the paper was in an active state, and dye angiography was not performed repeatedly.^[8]^ Therefore, it was inferred that angiography was performed when active CNV was suspected during patient follow-up. In contrast, in this study, even in cases with suspected clinical manifestations of active CNV, we performed OCTA to detect CNV, especially in patients having CSC with FIPED, which suggests the presence of type 1 CNV on OCT. Moreover, our results suggested that when OCTA was used together with FA and ICGA, the detection period of CSC could be shortened. The diagnosis of CSC can be challenging when it is complicated by CNV^[18,19]^ because the clinical features and presentation coexist in both conditions.^[20]^ The rate of CNV detection in patients with CSC varies from 24% to 42% among studies.^[10,12,20,21]^ It has been reported that in comparison with FA/ICGA, OCTA can detect CNV more efficiently when accompanied by flat irregular PED, which is an OCT finding suggesting type 1 CNV.^[10,22]^ We also found that CNV was detected earlier than in previous studies when OCTA was used together with FA and ICGA, highlighting the efficacy of the OCTA test in patients with CSC.

There are few reports in the literature regarding the long-term prognosis in patients with CSC complicated by CNV. However, it has been reported that CSC was associated with poor long-term visual prognosis,^[7,8]^ and that the presence of CNV was associated with lower baseline vision when data of patients with CSC with and without CNV was compared.^[11]^ In this study, we observed the patients with CSC for an average period of 33.79 ± 27.02 months. In a follow-up of more than 1 year after CNV detection, CMT decreased significantly. However, there was no statistically significant change in the BCVA after 5-year follow-up from CNV detection and the patients had stable and good vision.

This study included patients who received anti-VEGF treatment or PDT. Twenty four out of 30 eyes (80%) received at least 1 anti-VEGF therapy during follow-up. The result of decreased CMT during follow-up may be related to anti-VEGF and PDT therapy. Although it has been reported that the intraocular VEGF levels were not elevated in patients with CSC,^[23]^ anti-VEGF is considered to reduce the choroidal hyperpermeability and induce the reduction in SRF and has been used as a treatment in patients with chronic CSC.^[24]^ A pilot study was informative, as there were limited randomized study results on the effect of anti-VEGF therapy in CSC cases. The study used aflibercept in a patient with CSC and reported significant improvement in visual acuity and CMT after treatment.^[25]^ However, a randomized controlled study by Lim et al reported that there was no significant difference in visual acuity, central retinal thickness, and duration of SRF between bevacizumab treatment and observation in a patient with CSC.^[26]^ To date, the visual prognosis of CSC in patients with CNV and the effect of the treatment remain poorly understood. We demonstrated that there was no significant decrease in the visual acuity of patients with CSC and CNV during a 1-year follow-up in a real-world setting. This suggests that the visual prognosis was not bad at the 1-year follow-up, considering that the patients with CSC with CNV had relatively good vision at the time of CNV detection. No case was diagnosed with active wet ARMD during the follow-up in our study and there was no case with subretinal, intraretinal hemorrhage or drusen during follow-up. In a univariate analysis of risk factors associated with visual prognosis in a 5-year follow-up, the initial BCVA values were significantly correlated with visual prognosis; in contrast, no significant correlation was observed in multivariate analysis. Therefore, it is difficult to find a risk factor associated with visual prognosis because most patients in this study maintained relatively good vision after 5 years. Further studies with more cohorts are needed in the future.

The spontaneous resolution of the SRF and PED accompanying CSC is common; thus, this should be considered while deciding on treatment plans.^[1,27–29]^ However, cases of neovascular rather than non-neovascular CSC accompanied by CNV are more likely to require treatment, including photodynamic therapy or focal laser photocoagulation, to resolve SRF.^[14,30]^ In a previous study, intravitreal anti-VEGF therapy for neovascular CSC showed better anatomic results and visual outcomes compared to laser therapy, suggesting that it should be considered for patients with CSC complicated by CNV.^[31,32]^ Considering that CNV is associated with poor visual prognosis in patients with CSC,^[7,8]^ early detection of CNV by OCTA is crucial for patient monitoring and management planning. Our results showed that CNV is suspected when FIPED is observed on OCT in patients with CSC, and FA/ICGA and OCTA should be performed.

However, our study had some limitations. First, because of the retrospective design of the study, we could not ascertain the period of CNV development in patients with CSC. Our study was also limited by the relatively small number of patients and sample power at 3 and 5 years. Further prospective studies with larger sample sizes are needed to confirm our findings. Additionally, there was a possible time gap between actual CNV occurrence and the patient's visit for examination. In this study, there was no significant worsening of CMT and BCVA during the 5-year follow-up after CNV detection. However, considering that CSC is prevalent in middle-aged individuals, 5 years is a relatively short period to determine the final visual prognosis. Therefore, a long-term cohort study should be conducted in the future.

## 5 Conclusions

In conclusion, CNV was detected earlier in our study than in a previous study. Furthermore, after CNV detection, there was no rapid deterioration of visual acuity or clinical features in patients with CSC. Moreover, OCTA is useful for detecting inactive CNV when FIPED is found on OCT during follow-up of patients with CSC.

## Author contributions

**Data curation:** Gun-Jung Ma.

**Formal analysis:** Woo-Kyung Park, Mirinae Kim, Rae-Young Kim.

**Funding acquisition:** Young-Hoon Park.

**Investigation:** Gun-Jung Ma.

**Project administration:** Young-Hoon Park.

**Supervision:** Young-Gun Park, Young-Hoon Park.

**Validation:** Rae-Young Kim.

**Visualization:** Woo-Kyung Park, Mirinae Kim.

**Writing – original draft:** Rae-Young Kim.

**Writing – review & editing:** Rae-Young Kim, Young-Gun Park.
